# The unique effects of supporting beginning teachers’ psychological needs through learning communities and a teacher-mentor’s support: A longitudinal study based on self-determination theory

**DOI:** 10.3389/fpsyg.2022.859364

**Published:** 2022-10-25

**Authors:** Haya Kaplan

**Affiliations:** Kaye Academic College of Education, Be’er-Sheva, Israel

**Keywords:** autonomous motivation, beginning teachers, induction, learning community, mentoring, psychological needs, self-determination theory

## Abstract

The induction period is considered one of the most difficult in a teacher’s career. In Israel, support systems for beginning teachers (BTs) include a learning community (LC) and a mentoring process, over a 2-year period. The study was based on self-determination theory and examined how support for BTs’ psychological needs and exploration from the LC facilitator and teacher-mentor contributed to their functioning. The study was conducted over 2 years during which BTs participated in LCs and were accompanied by a teacher-mentor. Questionnaires were administered to Bedouin-Arab and Jewish teachers, 308 interns in the first year, and 205 new teachers in the second. Results of SEM analysis indicated that supporting BTs’ needs and exploration by LC facilitators in both years predicted their autonomous motivation, which in turn predicted positive feelings and satisfaction in the LC. The LC facilitators’ support also predicted the BTs’ autonomous motivation in teaching, which was also predicted by the teacher-mentors’ support. This in turn positively predicted the BTs’ sense of competence and self-actualization, and negatively predicted burnout. In both years, autonomous motivation mediated the association between the support the BTs received, and teacher outcomes in the LC and school. No significant differences were found between the two cultures. The effects found in the second year are above and beyond the effects of support from the LC facilitators and teacher-mentors in the first year. The study indicates the importance of combining multiple induction programs over time and highlights the importance of supporting BTs’ needs during the induction period.

## Introduction

The induction period refers to the transition from teacher education to the teaching profession and includes the first years in teaching in the education system. It is a period in which beginning teachers (BTs)^1^ construct their professional identity (Kaplan et al., 2016), acquire the necessary knowledge, skills, and abilities for teaching, and adapt to school culture (Thomas et al., 2019).

The literature in Israel and around the world presents a wealth of studies on the difficulties encountered by BTs (Gavish and Friedman, 2010; Mansfield et al., 2014; Flores, 2017). They face pedagogical, emotional, and social difficulties, and find it hard to adapt to the organizational culture in the school (Gavish and Friedman, 2010; De Neve and Devos, 2017). These difficulties manifest psychologically, they are bound up in negative feelings and a sense of burnout (Clandinin et al., 2015; Voss et al., 2017), and lead to impaired motivation (Kaplan, 2021a), and to teachers leaving the profession (Sperling, 2015).

Studies indicate the importance of support systems for BTs (Kutsyuruba et al., 2017; Thomas et al., 2019). The most widely acknowledged program worldwide is mentoring, which has been the subject of numerous studies and surveys (Ingersoll and Smith, 2004; Kutsyuruba et al., 2017). However, little research information is available on the specific contribution of other programs that have been developed to support BTs (Fresko and Nasser-Abu Alhija, 2014). Additionally, studies indicate that BTs who receive support from a number of sources benefit in aspects such as greater retention in the teaching profession (Smith and Ingersoll, 2004), sense of belonging to the school, sense of competence in teaching, autonomous motivation, and reduced sense of burnout (Mcdossi and Kaplan, 2019). However, research on the unique contribution of multiple induction programs and their joint examination is still minimal.

In Israel, support frameworks include participation in a professional learning community (LC) of BTs (interns and new teachers) for 2 years, during which each BT is assigned an experienced teacher-mentor in the school where they teach. Studies indicate the contribution of such programs to BTs adjustment in emotional, professional, and organizational aspects, and to improved teaching methods (Abu Ras, 2010; Fresko and Nasser-Abu Alhija, 2014). The present study is a longitudinal study (over 2 years) that examined the unique effects of support from the LC facilitators and teacher-mentors during the internship year and the first year after internship on the functioning of BTs. To date, these support frameworks have been examined separately.

Another unique aspect of the present study is the theoretical perspective on which it is based, namely self-determination theory (SDT) (Ryan and Deci, 2017), and its focus on the unique effects of support from LC facilitators and teacher-mentors for BTs basic psychological needs on their functioning as teachers. The research literature includes studies on BTs from an SDT perspective (e.g., Fernet et al., 2016), however, few studies have focus on the influence of teacher-mentors on BTs from this perspective (Kaplan and Israel, 2020, Unpublished manuscript;^2^ Burger et al., 2021).

The present study seeks to add to the literature by examining the contribution of two BT support programs, which operate in different contexts. The BTs meet with their mentors in the school, and participate in an LC in an academic college of education. Is there linkage between the effects of the support BTs receive in these contexts? Specifically, will support in the workshop context (LC) affect the functioning of the BTs in the school context? For instance, will a positive effect be found on autonomous motivation in teaching or sense of competence in teaching, and a negative effect on sense of burnout?

Does participation in LCs in the second year of the induction period uniquely contribute to BTs, above and beyond the effects of the LC in which they participated in their internship year? The present longitudinal study provides answers to these questions.

## Theoretical background

### The beginning teacher’s world

A BT’s first year in teaching is important in shaping their identity as a teacher (Kaplan et al., 2016). At the same time, it is also considered one of most difficult periods in a teacher’s career, and many teachers experience a sharp transition from the training stage to the working stage (Schmidt et al., 2017). They come to teaching imbued with a sense of mission, but their dreams and ideals quickly turn into a daily struggle for survival (Pillen et al., 2013; Arar and Masry-Harzallah, 2016). These feelings stem from the gap between school reality and the professional knowledge, sense of competence in teaching, vision, and values with which the teachers have equipped themselves during their training.

Beginning teachers face personal, professional, and ecological difficulties (Abu Ras, 2010). The personal aspect refers to perceptions of self (e.g., sense of professional competence), to emotionally coping with a complex reality, and insights regarding teacher role perception. In the professional aspect, BTs have to contend with new pedagogical knowledge, adapting teaching methods to different populations, and solving educational problems in the classroom, e.g., discipline problems and motivation. The ecological aspect refers to contending with the school’s organizational environment, the staff, and administrators. BTs have to acquaint themselves with policies, procedures, and rules, and contend with the expectations of experienced teachers, administrative staff, students, and parents. As a consequence of these difficulties, many teachers drop out of the education system, a troubling phenomenon in many countries around the world, including Israel (Shapira-Lishchinsky et al., 2019).

### Induction programs for beginning teachers

Induction programs differ from one another in aspects such as the program’s target population, duration, components, and who is leading and developing it, e.g., a school or academic institution. In some cases, they are a national or district initiative (Howe, 2006; Fresko and Nasser-Abu Alhija, 2014; Sela and Harel, 2018). The most widely used program is mentoring (Ingersoll and Smith, 2004; Kutsyuruba et al., 2017). The literature describes additional programs that have been developed around the world, e.g., internet support from peers and mentors that facilitates sharing materials associated with teaching (Herrington et al., 2006), participating in group workshops (Lazovsky and Zeiger, 2004), peer group mentoring with mentors and BTs, a program developed and implemented in Finland (Pennanen et al., 2017), and others.

In light of the above difficulties, the Israel Ministry of Education, in collaboration with the academic colleges of education, established induction programs that include participation in two LCs in workshop format at the college: one in the internship year, and the other in the year after internship. Participation in these LCs is compulsory. In their internship year, the interns also undergo an evaluation process as a condition for receiving a teaching license. Additionally, the BTs are mentored by a teacher-mentor in a dyadic mentoring model. The present article focuses on these LCs, as presented below.

### Learning communities of interns and new teachers

Professional learning communities of teachers are groups in which teachers come together to promote their professional learning. These LCs are based on trust, partnership, and shared vision and goals, and facilitate a critical examination of teaching processes, knowledge development, and new skills and practices (Fresko and Nasser-Abu Alhija, 2014). They promote the teachers’ professional development and contribute to their wellbeing (Owen, 2016).

In Israel, LCs of interns and new teachers operate in workshop format and led by professional facilitators who are faculty members from the academic institution. They are experienced group facilitators, who possess the skills for promoting an authentic reflexive dialogue in a safe place in which to process emotional and social processes. The facilitators are leading diverse activities to enhance a positive climate in the group (Fresko and Nasser-Abu Alhija, 2014). The LCs are funded by the Ministry of Education and take place in colleges of education in collaboration with the Ministry of Education.

In the LCs, the teachers are expected to share their experiences with others. They discuss a variety of issues and professional dilemmas, learn about themselves as teachers, connect theory to practice, and construct their professional identity (Fresko and Nasser-Abu Alhija, 2014; Hammer-Budnaro, 2018). Each LC comprises 18–20 teachers. During the internship year, the groups meet once a week for 90 min throughout the academic year (60 h). In the year after internship, the BTs meet ten times during the year (30 h). These meetings are held independently of the teacher-mentors.

Studies in Israel indicate the effectiveness of the intern LCs, e.g., adjustment in emotional, professional, and organizational aspects (Abu Ras, 2010), the teachers’ satisfaction with the absorption processes (Lazovsky and Zeiger, 2004), increased self-confidence, and improved ability to contend with their students (Arviv-Elyashiv and Lederer, 2011). Fresko and Nasser-Abu Alhija (2014) found that the LCs (called “seminars” in the article) supported the teachers emotionally and enabled them to process their experiences without fear of being judged. In the beginning of the year, the teachers mainly focused on discipline problems, and, as the year progressed, on teaching processes, evaluation, and professional identity.

In Israel, the BTs dropout rate has declined over the years, especially among teachers who participated in intern LCs [Central Bureau of Statistics, Israel (CBS), 2019]: the dropout rate of teachers who did not undergo internship was up to 19% after 1 year, and up to 26.6% after 3 years, whereas the dropout rate of teachers who underwent internship and participated in an LCs was up to 8% after 1 year, and up to 11.3% after 3 years.

Additional studies, albeit fewer, indicated the effectiveness of the BT workshops 1 year after internship (Kaplan et al., 2016; Kaplan, 2021b). For example, Kaplan (2021b) found that teachers’ need support by workshop facilitators predicted sense of competence and autonomous motivation in the workshops, which in turn predicted autonomous motivation in teaching. Autonomous motivation in teaching was also predicted by the teacher-mentors’ need support, and in turn predicted teachers’ sense of competence in teaching, investment in school, and sense of self-actualization. This study served as the basis for the present longitudinal study, which examined the contributions of the LCs in the internship year and 1 year after internship among the same teachers.

### Self-determination theory and teacher motivation

According to SDT, people have three universal basic psychological needs: the need for relatedness, the need for sense of competence, and the need for autonomy. Satisfaction of these needs, by means of support from the environment, contributes to optimal development, autonomous motivation, sense of wellbeing, quality engagement, and social adjustment, as well as processes of integrating and internalizing behaviors and values, i.e., identity construction processes (Deci and Ryan, 2000; Reeve, 2006; La Guardia, 2009; Kaplan and Assor, 2012; Ryan and Deci, 2017, 2020; Kaplan, 2018).

The need for relatedness is the person’s striving to maintain close, secure, and satisfying relationships with others in their social environment, to be part of a community. The need for a sense of competence is the person’s striving to experience themselves as being able to realize plans, aspirations, and objectives, which are not always easy to attain, and to feel a sense of efficacy. The need for autonomy is the person’s striving for self-determination, authentic self-expression, meaning, independence, and freedom of choice (Deci and Ryan, 2000).

Motivation is a unique concept in SDT. It emphasizes the quality of motivation, and therefore refers to different motivation types, which are classified according to the person’s level of self-determination, i.e., the degree to which they feel that their activity is based on and emerges from their authentic inner desires (Deci and Ryan, 2000; Ryan and Deci, 2020). Autonomous motivation is a state wherein the person experiences a sense of choice, will, and self-determination, and acts from identification with the value or behavior (identified motivation), or from inner interest and profound satisfaction (intrinsic motivation). Controlled motivation is a state wherein the person acts from a sense of coercion, pressure, hope for reward or fear from punishment (extrinsic motivation), or from inner pressure, feelings of shame and guilt, and a desire to gain internal or external appreciation (introjected motivation).

Behaviors stemming from extrinsic motivation can become self-determined through a process of internalization (Deci and Ryan, 2000), which leads to the person perceiving the action as consistent with their identity, and as important in relation to other actions (integrative/autonomous motivation).

The SDT approach is based on research (Ryan and Deci, 2017) that has been conducted also in Israel (e.g., Roth et al., 2007; Kaplan, 2018; Kaplan and Assor, 2019). Extensive SDT-based research in the field of education has largely focused on the effects of teacher behaviors in supporting or suppressing students’ needs, and on student outcomes (Ryan and Deci, 2017). SDT also focuses on teachers’ motivation and can be generally classified into three populations: in-service teachers, pre-service teachers, and – the present study’s focus – BTs.

Support for the psychological needs of in-service teachers, and experiences of need satisfaction, have been found to be associated with positive outcomes, e.g., autonomous motivation in teaching (Katz and Cohen, 2018), and an autonomy and competence supportive teaching style (Moè and Katz, 2020). Teachers’ autonomous motivation was found to be associated with outcomes such as investment in work (in de Wal et al., 2014), job satisfaction and wellbeing (Nie et al., 2014), sense of self-actualization, and student autonomy support (Roth et al., 2007; van den Berghe et al., 2014). Conversely, experiences of need suppression were found to predict controlled motivation and a range of negative outcomes among teachers (Vansteenkiste and Ryan, 2013).

Other SDT-based studies have focused on pre-service teachers (e.g., Evelein et al., 2008; Wang and Liu, 2008; Bouwma-Gearhart, 2010; Perlman, 2015; Kaplan and Madjar, 2017). For example, Kaplan and Madjar (2017) found that supporting the needs of Jewish and Bedouin Arab pre-service teachers contributed positively to predicting sense of relatedness, sense of competence, and autonomous motivation. Autonomous motivation contributed positively to predicting self-actualization, investment, and self-exploration, while controlled motivation predicted emotional burnout.

SDT-based studies on BTs are comparatively few (e.g., Fernet et al., 2016; Kaplan et al., 2016; Kaplan, 2021b). For example, Fernet et al. (2016) found that receiving a positive work evaluation is associated with autonomous motivation, which in turn predicts commitment to work, positive relationships with students, and reduced emotional burnout, while workload predicts controlled motivation, which in turn predicts emotional burnout.

### Exploration support as means of need support

A need supportive environment, which constitutes a safe space for exploration, promotes BTs’ processes of autonomous identity construction (Kaplan et al., 2016). The term “exploration” refers to a process wherein a person engages in inquiry and a reflective search for meaningful information for their development (Flum and Kaplan, 2012). Exploration is conducted around feelings, beliefs, values, personal goals, vision, actions and future perceptions, and also focuses on gaps that emerge between what the teacher brings with them to teaching, and the reality they encounter. These aspects are associated with the need for autonomy, and this is a process that is characteristic of BTs. Consequently, exploration support is a way of supporting BTs’ autonomy (Kaplan et al., 2016). Examination of practices associated with processes of identity construction and exploration among school students indicates that they are ways of supporting autonomy (Assor et al., 2002; Reeve, 2013). This argument is supported by Assor’s definition of the need for autonomy as “the striving to form and realize authentic and direction-giving values, goals, and interests (i.e., the striving for an inner compass)” (Assor, 2012, p. 421; Kaplan and Assor, 2019).

One way to promote an inner compass is by means of reflective inner compass facilitation, i.e., supporting the examination of values/goals/interests (e.g., Assor, 2012; Kaplan and Assor, 2019). The present study incorporates exploration support as a way of supporting BTs’ autonomy.

### The teacher-mentor’s support and Its contribution

The role of teacher-mentors and their positive effects on the induction process have been extensively researched and reported in the professional literature (Howe, 2006; Wang and Odell, 2007; Hennissen et al., 2008; Ingersoll and Strong, 2011; Kutsyuruba et al., 2017; Alegado and Soe, 2021; Ewing, 2021). The mentors accompany the BTs in professional, emotional, social, and organizational aspects, provide feedback, and, in Israel, also evaluate them (Ford, 2017).

The mentor’s support was found to be associated with various outcomes, e.g., BTs’ sense of professional efficacy (LoCasale-Crouch et al., 2012), their sense of wellbeing, and teacher enthusiasm (Richter et al., 2013), improved teaching practices (Alegado and Soe, 2021), job satisfaction, reduced emotional burnout (Richter et al., 2013), motivation (Kaplan, 2021b), and persistence in the profession (Rots et al., 2007).

The professional literature distinguishes between different paradigms of mentoring (Cochran-Smith and Paris, 1995; Feiman-Nemser, 2001; Richter et al., 2013; Burger et al., 2021). The traditional paradigm of conventional or transmission-oriented mentoring is based on behavioral learning theories. The mentor is perceived as an authority, and the mentoring style is one of conveying knowledge, practices, skills, and pedagogical emphases (e.g., how to manage a classroom, how to build lessons), which the mentor (or the school) perceives as suitable from a professional point of view. Another paradigm focuses on knowledge transformation and is also known as educative mentoring or constructivist-oriented mentoring. This style of mentoring emphasizes a collaborative relationship between mentor and mentee, and knowledge is created jointly in processes that promote growth, inquiry, and learning from practice. The mentoring relationship is not hierarchic, and the mentoring is based on joint reflection and autonomous decision-making (Burger et al., 2021).

Studies on BTs found that the constructivist approach, in comparison to the transmission model approach, leads to more positive outcomes among BTs, including sense of competence, teacher enthusiasm, job satisfaction, and lower levels of burnout (Richter et al., 2013; Voss et al., 2017), as well as reduced levels of emotional burnout by supporting the need for autonomy among mentees (Burger et al., 2021).

The humanistic approach, which underlies the constructivist paradigm, presents the desirable mentoring relationship according to SDT (Orland-Barak and Wang, 2020). The research literature describing the connection between mentoring and SDT refers to varied and diverse populations, including school students and teachers (e.g., Dantzer, 2017), higher education students and faculty (e.g., Lechuga, 2014), and to the workplace (e.g., Kennett and Lomas, 2015). Studies on the effects of mentoring on a population of BTs from an SDT perspective are few and far between (Kaplan and Israel, 2020, Unpublished manuscript, See footnote 2; Burger et al., 2021). Consequently, the present study contributes to the research literature in this regard.

### The cultural perspective of the present study

The participants in the present study were interns and new teachers from two cultural groups: Jewish teachers (individualistic culture) and Bedouin Arab teachers (collectivist culture). This multicultural profile is commonplace in colleges of education in Israel, and therefore it is important to examine the differences (if any) between the motivational processes in these two cultural groups.

The study was conducted in a college of education attended by Jewish and Bedouin Arab students. The Jewish students in the specific college, belong to a secular culture that can be defined as individualistic, while the Bedouin Arab students belong to a culture that can be defined as hierarchical-collectivist (Hofstede et al., 2010) and patriarchal (Sharabi, 2014; Arar and Masry-Harzallah, 2016). The unique social structure of Bedouin society is based on tribalism and characterized by loyalty to the membership group (family, tribe), adherence to honor values, rigid hierarchical structures, and a high level of obedience to male-parental authority (Alsayed, 2015). While changes have been taking place in Bedouin society in recent decades, it can still be defined as hierarchical-collectivist and patriarchal (Oplatka and El-Kuran, 2020; Zoabi and Abed-Said, 2021). The schools reflect the society and its characteristics, relationships between teachers resemble relationships between family members, and generally employ traditional, frontal teaching methods (Alian, 2013).

Researchers advocating a cultural relativism approach claim that the need for autonomy is a Western ideal and is not important in traditional cultures, which emphasize conformity, obedience, and co-dependency within the family (Iyengar and DeVoe, 2003), and, in the present case, dependency on authority figures in the school. According to this approach, it could be argued that Bedouin Arab BTs have internalized the patterns of communication and relationships in Bedouin society, and, consequently, autonomy support or suppression will not have a marked effect on them. According to SDT, psychological needs are universal and innate (Deci and Ryan, 2000). Previous studies have indicated the theory’s applicability in collectivist cultures as well (e.g., Jang et al., 2009; Ginevra et al., 2015), including Bedouin society in Israel (Kaplan and Israel, 2020, Unpublished manuscript, See footnote 2).

However, previous studies in Bedouin society that examined need satisfaction among teachers, primarily focused on in-service teachers (e.g., Katz and Cohen, 2018), and pre-service teachers (e.g., Kaplan and Madjar, 2017). The present study constitutes a continuation of previous studies that examined motivational processes among Bedouin Arab teachers, and focuses on teacher interns and new teachers. This specific research population of Bedouin Arab BTs has gained relatively little research attention from an SDT perspective (e.g., Kaplan, 2021a,b). The present study also examines the perceptions of BTs regarding their teacher-mentors. SDT-based research on mentors of BTs in general, and Bedouin Arab BTs in particular, is very limited (Kaplan and Israel, 2020, Unpublished manuscript, See footnote 2; Burger et al., 2021).

### The present study and its importance

The present study is a 2-year longitudinal study. In the first year, the participants were interns who taught in schools, but were still fourth-year college students, and participated in an intern LC. In the second year, the participants were teachers who had completed their internship year and participated in a new-teacher LC. Each year these LCs were led by a different facilitator.

**Figure 1:**
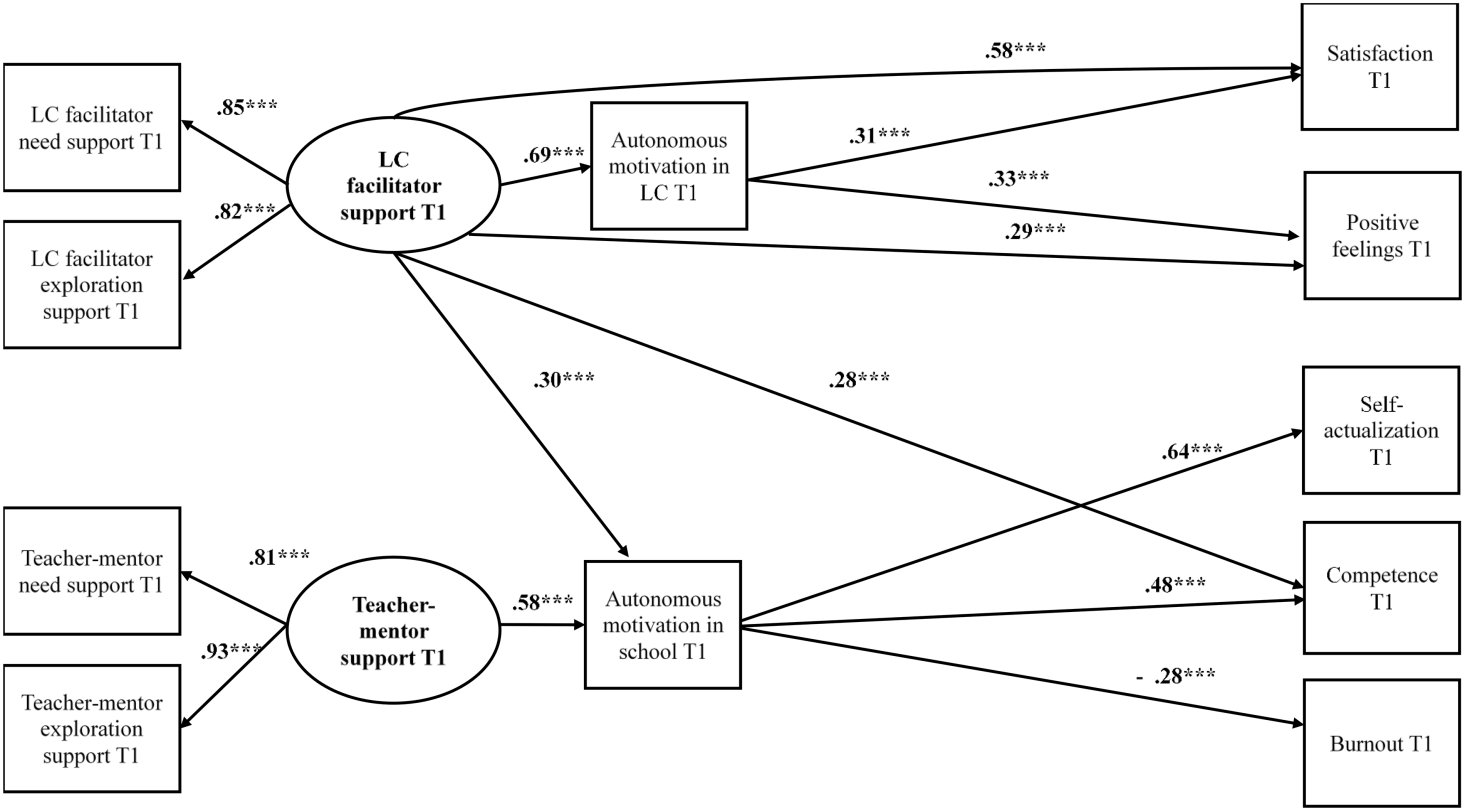
Results for the hypothesized structural model – T1. ***p<0.001.

The study examined the unique effects of need support and exploration support from different figures on the functioning of BTs in the LCs and the school (see Table 1). The BTs received support from the following figures: (1) The intern LC facilitator; (2) the new-teacher LC facilitator; and (3) a teacher-mentor throughout the 2 years. The research questionnaires were administered to the interns, and the following year to the new teachers who had participated in the intern LC the year before. The study enables an examination of the unique contribution of two induction programs that the BTs experienced simultaneously over a 2-year period. The support they received and their functioning in the LC and the school in each of the 2 years were examined by means of the variables presented in table.

**Table 1:** The research variables.

LC (in the college)	School
Psychological need support and exploration support from the LC facilitator	Psychological need support and exploration support from the teacher-mentor
Autonomous motivation in the LC	Autonomous motivation in teaching
Positive feelings	Self-actualization
Sense of satisfaction	Sense of competence in teaching
	Sense of burnout

The contribution of the LC facilitators’ and mentors’ support was examined by means of an SDT mediation model whereby psychological need support predicts autonomous motivation, which in turn predicts various positive outcomes (Ryan and Deci, 2017).

The outcome variables were matched to the context. In the workshop (LC) context, where it is the facilitator who supports the participants, the outcome variables that can be tested are autonomous motivation in the workshop (mediating variable), and sense of satisfaction and positive feeling regarding the workshop. In the school context, where it is the mentor who supports the participants, the outcome variables that can be tested are autonomous motivation in teaching, and variables associated with optimal functioning in school: self-actualization, sense of competence in teaching, and sense of burnout.

In the present study, the model was simultaneously tested in two contexts (the college and the school), which have been examined separately until now. This research design enables examination of the contribution of the LC facilitators’ support to the BTs’ motivation in school (not only in the LC). Since the LCs engage with relevant issues pertaining to the school and teaching, it may be assumed that the facilitators’ support will be found to have an effect in the school context as well.

The study joins previous studies that examined the SDT mediation model by means of a longitudinal design (e.g., Jang et al., 2012, 2016; Oga-Baldwin et al., 2017). This enabled the researcher to examine whether the support provided by the LC facilitators and mentors in the second year has a unique contribution beyond the support the BTs received in the first year. It could be argued that the various outcomes found in the study’s second year would be false associations. In other words, common influences would be found in the second year with the teachers’ autonomous motivation and sense of self-actualization, sense of competence in teaching, and burnout in the first year; and the LC facilitators’ and mentors’ support in the second year would not contribute beyond what the BTs bring with them to the LC in the second year. Thus, for example, teachers might come with high autonomous motivation to participate in the new-teacher LC (due to the facilitator’s support during the internship year), and their motivation the year after internship will reflect that initial motivation, and not the unique contribution of the LC facilitator’s support in the second year. This kind of finding could provide evidence of the ineffectiveness of the LC in the second year, a highly important finding considering the time the teachers devote to participating in the LCs, as well as economic considerations.

### Research hypotheses

The interns’ autonomous motivation in the LC (T1) will mediate the association between the LC facilitator’s support for their basic needs and exploration (T1), and their satisfaction and positive feelings in the LC (T1). Additionally, the interns’ autonomous motivation in teaching (T1) will mediate the association between the LC facilitator’s and teacher-mentors’ support for their basic needs and exploration (T1), and their sense of competence, self-actualization, and burnout (T1).

The new teachers’ autonomous motivation in the LC (T2) will mediate the association between the LC facilitator’s support for their basic needs and exploration (T2), and their satisfaction and positive feelings in the LC (T2). Additionally, the new teachers’ autonomous motivation in teaching (T2) will mediate the association between the LC facilitator’s and teacher-mentors’ support for their basic needs and exploration (T2), and their sense of competence, self-actualization, and burnout (T2), above and beyond the variance of the mediating and dependent variables at T1.

In both models no differences will be found between Bedouin Arab and Jewish interns.

## Materials and methods

### Participants

The participants at T1 were 308 BTs in their internship year who were participating in intern LCs. The internship year is the fourth year of the B.Ed. program. During the year, the students complete their academic studies, and intern as teachers in schools prior to receiving their teaching license. The interns belong to different cultures, i.e., Jews and Bedouin Arabs, and come from a range of specialties, e.g., special education, Arabic, literature, mathematics, physical education, etc. They participate in intern LCs, and most of them go on to participate in new-teacher LCs (the year after internship). Thus, students from all the subjects taught at the college participated in the same LC. The interns’ age ranged from 19 to 40 (*M* = 25.43, *SD* = 4.40). The sample consisted of 35 men (11.4%) and 273 women (88.6%); 152 were Jewish (49.3%), and 156 (50.7%) were Bedouin Arabs. They taught in a variety of educational settings: kindergarten (32.2%), elementary school (48.5%), and junior high and high school (19.3%).

The participants at T2 were 205 new teachers in their first year of teaching who have a teaching license (having completed their internship year) and are working as teachers. These teachers were interns the year before, and participated in the internship workshops.

Only participants who completed questionnaires at both measurement times were included in the study. The new teachers’ age ranged from 20 to 41 (*M* = 26.46, *SD* = 4.40). The sample consisted of 23 men (11.2%) and 182 women (88.8%); 91 were Jewish (44.4%) and 114 were Bedouin Arabs (55.6%). They taught in a variety of educational settings: kindergarten (29.4%), elementary school (44.4%), and junior high and high school (26.2%).

### Tools

The research tools used in the study were validated in previous studies of teacher populations (Roth et al., 2007; Kaplan and Madjar, 2017), and adapted to the present study. The responses in the various questionnaires ranged on a scale of 1–5 from “Strongly agree” (5) to “Strongly disagree” (1). The following is a description of the research tools.

### Perceptions of the LC facilitators’ and teacher-mentors’ need supportive behaviors

The scale is based on previous studies (Assor et al., 2002; Kaplan et al., 2014), and was developed and validated for a teacher population (Roth et al., 2007; Kaplan and Madjar, 2017). The questionnaire consisted of 24 items, which included support for relatedness (e.g., “The facilitator/teacher-mentor gives the participants a feeling of trust and security”), support for competence (e.g., “The facilitator/teacher-mentor bolsters my faith in my ability as a teacher”), and support for autonomy that also included support for choice (e.g., “The facilitator/teacher-mentor encourages me to work in my own way”), and encouraging relevance (e.g., “It is important to the facilitator/teacher-mentor to hear what each person in the group thinks, even if it’s different from other opinions or their own opinion”). Variables were calculated by averaging all the items, with high scores indicating high levels of need support (LC facilitator need support: T1 α = 0.95, T2 α = 0.84; teacher-mentor need support: T1 α = 0.96, T2 α = 0.95).

### Exploration support from the LC facilitators and teacher-mentors

The exploration-support scale was built in accordance with the perception that autonomy support also includes support for the formulation of values, and providing opportunities for the teachers to deepen their familiarity with themselves: What kind of teacher do I want to be? What are my strengths and outlooks? and to clarify important questions that occupy them. The questionnaire was validated in previous studies, and found to be reliable (Assor, 2010, 2012), and was adapted for the present study. Examples from the 5-item questionnaire: “The workshop sessions enable me to understand what kind of teacher I want to be”; “The discussions in the workshop sessions enable me to examine how to act correctly and appropriately in matters that are important to me in my work.” Variables were calculated by averaging all the items, with high scores indicating high levels of exploration support (LC facilitator exploration support: T1 α = 0.85, T2 α = 0.87; teacher-mentor exploration support: T1 α = 0.86, T2 α = 0.84).

### Autonomous motivation in the LC and in teaching

The questionnaire was developed and validated by Ryan and Connell (1989). It has been applied in numerous studies in the field of education (Assor et al., 2002; Ryan and Deci, 2017), and adapted to teachers (Roth et al., 2007). The 12-item questionnaire included subscales of identified motivation (e.g., “I invest efforts in the workshop because in the sessions, I learn what it is to be a teacher / I invest effort in teaching, because that way I can achieve better results as a teacher”), and intrinsic motivation (e.g., “I invest effort in the workshop because the subjects we talk about interest me / I invest in teaching because being a teacher interests me”). Variables were calculated by averaging all the items, with high scores indicating high levels of autonomous motivation in the LC and in teaching (autonomous motivation in the LC: T1 α = 0.97, T2 α = 0.95; autonomous motivation in teaching: T1 α = 0.86, T2 α = 0.86).

### Satisfaction with the LC

The questionnaire examined the BTs’ satisfaction with the LC, the learning they experienced, the content, and the processes. “Satisfaction” is an accepted variable in the study of novice teachers (Ilaiyan and Zedan, 2011). The 7-item questionnaire included, for example: “If I could choose again, I would choose this workshop again.” The variable was calculated by averaging all the items, with high scores indicating high levels of satisfaction with the LC (T1 α = 0.93, T2 α = 0.89).

### Positive feelings in the LC

The scale refers to the participants’ positive feelings in the LC, and is based on previous research (Assor et al., 2002). The 10-item questionnaire included, for example: “I felt comfortable in the sessions.” The variable was calculated by averaging all the items, with high scores indicating high levels of positive feelings in the LC (T1 α = 0.93, T2 α = 0.89).

### Sense of competence in teaching

The questionnaire examined the participants’ self-reported perceptions of contending with challenges and difficulties in teaching. Based on previous studies (Assor et al., 2002; Kaplan and Madjar, 2017), the 5-item questionnaire included, for example: “When I decide to perform a difficult task related to teaching, I can do it.” The variable was calculated by averaging all the items, with high scores indicating high levels of sense of competence in teaching (T1 α = 0.80, T2 α = 0.73).

### Self-actualization as opposed to teacher burnout

These two scales are based on a shortened version of a scale used by Friedman and Farber (1992). The self-actualization variable reflects satisfaction with teaching, vitality, and energy, and a sense that teaching enables actualization of individual abilities. According to SDT, satisfying the need for autonomy entails authentic self-actualization (Deci and Ryan, 2000). The “sense of burnout” variable expresses a sense of depleted emotional and cognitive resources, a feeling of overload and stress (Maslach and Jackson, 1981), and of not being sufficiently or intensively involved in their work (Roth et al., 2007; van den Berghe et al., 2014). Both scales were validated in previous studies of teachers, and adapted to BTs (Roth et al., 2007; Eyal and Roth, 2011; van den Berghe et al., 2014). The 5-item self-actualization questionnaire included, for example: “I feel that teaching allows me to realize my full potential.” The 6-item burnout questionnaire included, for example: “I feel very tired in the morning when I have to go out to another day of work in school.” Variables were calculated by averaging all the items, with high scores indicating high levels of self-actualization and burnout in school (self-actualization: T1 α = 0.82, T2 α = 0.80; burnout T1 α = 0.78, T2 α = 0.81).

## Procedure

The questionnaires were administered over a 2-year period: the participants in the first year were interns, and in the second year they were new teachers. The questionnaires at both time measurements were in Hebrew (since the sessions were conducted in Hebrew), and administered by Hebrew-and Arabic-speaking research assistants without the presence of LC facilitators. Participants were given an explanation of the study’s goals, and could choose not to complete the questionnaires. Informed consent was obtained from all the participants included in the study. Confidentiality was guaranteed. It took approximately 45 min to complete the questionnaire.

In the first year (T1), the participants were asked to write down the last four numerals of their ID number on the questionnaire to enable us to pair the two questionnaires in the second year (T2). The participants received an explanation concerning this procedure, and assurance of confidentiality despite the identifying detail. The method of analysis was explained to them, which does not enable the person analyzing the information to know who completed the questionnaire. Only a few participants chose not to complete the questionnaire. The participants completed the questionnaire in the second half of the second semester of their participation in the LC, in most cases in the seventeenth or eighteenth session. The new teachers (T2) completed the questionnaires in the eighth session (out of ten).

## Analyses

Univariate and multivariate outliers were examined. No abnormality was observed. Thus, all the participants were included in the data analyses. Then, the means, standard deviations, and correlations between the research variables were examined separately at each measurement time. Then, the hypothesized mediation models were tested by means of structural equation modeling (SEM), using AMOS 21 software. Following Hoyle and Panter (1995), the model’s fit to the data was evaluated using five goodness of fit indices. Two of these indices were absolute: the χ2 statistic, and the Root Mean Square Error of Approximation (RMSEA). The remaining three indices were incremental: the Normed Fit Index (NFI), the Comparative Fit Index (CFI), and the Tucker-Lewis Index (TLI). A RMSEA below 0.06 in combination with NFI and CFI above 0.95 indicates excellent fit, whereas values below 0.08 and above 0.90, respectively, indicate adequate fit.

The significance of the mediation model at T1 was investigated. Then, the significance of the mediation model at T2 was investigated, followed by controlling for the variance of the variables at T1. The indirect effects were examined by means of bootstrapping analyses, using 10,000 resamples with a 95% confidence level to test the mediation hypotheses. An indirect effect coefficient was considered significant when the confidence interval (CI) values did not include zero. Additionally, multigroup analyses were conducted to investigate whether the mediation model is equivalent for Bedouin Arab and Jewish students at both measurement times.

## Results

### Preliminary analyses

Descriptive statistics and correlations between research variables were conducted separately for each measurement time (see Tables 2, 3). In addition, correlations between the research variables at T1 and T2 were calculated (see Table 4).

**Table 2:** Descriptive statistics and correlations between the research variables at T1.

**Variable**	**M (SD)**	**2**	**3**	**4**	**5**	**6**	**7**	**8**	**9**	**10**	**11**
1. LC facilitator need support	4.50 (0.48)	0.75**	0.45**	0.40**	0.49**	0.54**	0.62**	0.38**	0.61**	0.40**	−0.22**
2. LC facilitator exploration support	4.31 (0.61)		0.36**	0.43**	0.67**	0.54**	0.75**	0.48**	0.53**	0.40**	−0.17*
3. Teacher-mentor need support	4.43 (0.75)			0.70**	0.28**	0.64**	0.41**	0.27**	0.49**	0.49**	−0.21**
4. Teacher-mentor exploration support	4.20 (0.86)				0.34**	0.60**	0.42**	0.30**	0.39**	0.41**	−0.10
5. Autonomous motivation in the LC	4.20 (0.60)					0.45**	0.71**	0.53**	0.34**	0.38**	−0.12
6. Autonomous motivation in teaching (school)	4.44 (0.54)						0.51**	0.39**	0.66**	0.64**	−0.18**
7. Sense of satisfaction in the LC	4.15 (0.74)							0.52**	0.41**	0.38**	−0.26**
8. Positive feelings in the LC	3.58 (0.95)								0.31**	0.27**	−0.14
9. Sense of competence in teaching (school)	4.58 (0.45)									0.44**	−0.22**
10. Self-actualization	2.44 (1.42)										−0.37**
11. Burnout	4.18 (0.74)										

*p < 0.05; **p < 0.001.

**Table 3:** Descriptive statistics and correlations between the research variables at T2.

Variable	M SD	2	3	4	5	6	7	8	9	10	11
1. LC facilitator need support	4.49 (0.39)	0.66**	0.55**	0.49**	0.34**	0.42**	0.58**	0.39**	0.50**	0.42**	−0.28**
2. LC facilitator exploration support	4.35 (0.63)		0.29**	0.32**	0.66**	0.28**	0.69**	0.51**	0.32**	0.36**	-0.17**
3. Teacher-mentor need support	4.44 (0.49)			0.74**	0.09	0.51**	0.26**	0.23**	0.54**	0.48**	−0.18**
4. Teacher-mentor exploration support	4.30 (0.61)				0.16*	0.43**	0.32**	0.28**	0.46**	0.46**	−0.26**
5. Autonomous motivation in the LC	4.15 (0.77)					0.28**	0.65**	0.61**	0.16*	0.32**	−0.13*
6. Autonomous motivation in teaching (school)	4.44 (0.50)						0.25**	0.21**	0.59**	0.66**	−0.24**
7. Sense of satisfaction in the LC	4.31 (0.62)							0.59**	0.32**.	0.39**	−0.25**
8. Positive feelings in the LC	3.60 (0.70)								0.17**	0.27**	−0.17*
9. Sense of competence in teaching (school)	4.55 (0.42)									0.58**	−0.19**
10. Self-actualization	4.15 (0.59)										−0.39**
11. Burnout	2.58 (0.81)										

*p < 0.05; **p < 0.001.

**Table 4:** Correlations between the research variables at T1 and T2.

Variable	Variables at T1										
	1	2	3	4	5	6	7	8	9	10	11
1.LC facilitator need support	0.38**	0.25**	0.34**	0.27**	0.18*	0.38**	0.19*	0.23*	0.35**	0.370**	−0.144
2.LC facilitator exploration support	0.20*	0.26**	0.13	0.17	0.27**	0.24**	0.14	0.24**	0.23*	0.30**	−0.03
3.Teacher-mentor need support	0.39**	0.28**	0.58**	0.55**	0.18*	0.53**	0.27**	0.25**	0.41**	0.38**	−0.07
4.Teacher-mentor exploration support	0.34**	0.31**	0.46**	0.44**	0.26**	0.42**	0.22*	0.29**	0.28**	0.37**	0.07
5.Autonomous motivation in the LC	0.12	0.28**	0.01	0.09	0.43**	0.16	0.22*	0.28**	0.14	0.14	0.05
6.Autonomous motivation in teaching	0.45**	0.38**	0.44**	0.37**	0.20*	0.60**	0.25**	0.16	0.40**	0.41**	−0.12
7.Sense of satisfaction in the LC	0.28**	0.22*	0.18*	0.22*	0.23**	0.27**	0.27**	0.30**	0.21*	0.26**	−0.07
8.Positive feelings in the LC	0.17*	0.20*	0.08	0.06	0.23*	0.24**	0.14	0.41**	0.18*	0.25**	−0.05
9.Sense of competence in teaching	0.33**	0.27**	0.36**	0.26**	0.19*	0.53**	0.17	0.11	0.52**	0.35**	−0.09
10.Self-actualization	0.39**	0.38**	0.45**	0.38**	0.144	0.60**	0.22*	0.173	0.48**	0.59**	−0.16
11.Burnout	−0.40**	−0.30**	−0.21*	−0.121	−0.14	−0.30**	−0.30**	−0.29*	−0.37**	−0.39**	0.24**

*p < 0.05; **p < 0.01.

The findings presented below refer to both measurement times, and between times. Positive correlations were found between the BTs’ need support in the LC (from the facilitator) and the school (from the teacher-mentors) and autonomous motivation in the LC and the school, satisfaction with the LC, positive feelings in the LC, sense of self-actualization, and sense of competence in teaching, and a negative correlation with sense of burnout in teaching. Additionally, positive correlations were found between the BTs’ exploration support in the LC (from the facilitator) and the school (from the teacher-mentor) and autonomous motivation in the LC and the school, satisfaction with the LC, positive feelings in the LC, sense of self-actualization, and sense of competence in teaching, and a negative correlation with sense of burnout in teaching. Also, positive correlations were found between the BTs’ autonomous motivation in the LC and the school, and satisfaction with the LC, positive feelings in the LC, sense of self-actualization, and sense of competence in teaching, and a negative correlation with sense of burnout in teaching.

### Main analyses

Although T2 included control for the variance in T1, it was important to present the mediation model of T1 in the present study. This is because during the 2 years of the research, different people served as mentors and LC facilitators. Thus, in order to show the effect of continued support of both the teacher-mentor and LC facilitator, the associations at T1 are presented first, followed by the associations at T2, beyond the support provided at T1.

### Testing hypothesis 1: Mediation model T1

To examine the first research hypothesis (H1), the hypothesized mediation model was tested by means of structural equation modeling (SEM), using AMOS 21 software. The model includes the variables “LC facilitator need and exploration support” (T1) and “teacher-mentor need and exploration support” (T1) as independent exogenous latent variables. The variables “autonomous motivation in the LC” (T1), and “autonomous motivation in school” (T1) are mediating variables, while the variables “satisfaction in the LC” (T1), “positive feelings in the LC” (T1), “self-actualization” (T1), “sense of competence in teaching” (T1), and “sense of burnout in teaching” (T1), are dependent variables.

First, the goodness of fit of the model to the data was tested. The results show goodness of fit between the hypothesized model and the data: χ^2^ = 75.71, df = 35, *p* = 0.03; NFI = 0.94; TLI = 0.94; CFI = 0.97; RMSEA = 0.06. Then, the associations in the hypothesized model were examined. Figure 1 shows the results.

The analysis results show that the structural model and the anticipated associations were confirmed. A significant positive association was found between the LC facilitator’s support for the interns and their autonomous motivation in the LC (β = 0.69, *t* = 10.41, *p* < 0.001), which in turn predicted their satisfaction with the LC (β = 0.31, *t* = 5.00, *p* < 0.001), and their positive feelings in the LC (β = 0.33, *t* = 3.92, *p* < 0.001). Additionally, a direct significant positive association was found between the LC facilitator’s support for the interns and their satisfaction with the LC (β = 0.58, C.R. = 8.22, *p* < 0.000), and their positive feelings in the LC (β = 0.29, *t* = 3.33, *p* < 0.001). Consequently, and in accordance with H1, the interns’ autonomous motivation in the LC was found to partially mediate the association between the facilitator’s support for the interns and their satisfaction [indirect effect = 0.21, CI (0.08, 0.48)] and with their positive feelings in the LC (indirect effect = 0.23, CI [0.10, 0.44]).

It further emerges from the analysis that the facilitator’s support (β = 0.30, *t* = 4.30, *p* < 0.001) and the teacher-mentor’s support (β = 0.58, *t* = 7.71, *p* < 0.001) for the interns significantly and positively predicted their autonomous motivation in school, which in turn predicted their level of self-actualization (β = 0.64, *t* = 11.70, *p* < 0.001), sense of competence in teaching (β = 0.48, *t* = 7.38, *p* < 0.001), and sense of burnout in school (β = −0.28, *t* = −4.08, *p* < 0.001). Additionally, a direct significant positive association was found between the LC facilitator’s support and the interns’ sense of competence in teaching (β = 0.28, *t* = 4.38, *p* < 0.001). Consequently, and in accordance with H1, the interns’ autonomous motivation in school was found to fully mediate the association between the LC facilitator’s support and their self-actualization (indirect effect = 0.19, CI [0.08, 0.26]). However, the interns’ autonomous motivation in school did not mediate the association between the LC facilitator’s support and their sense of burnout (indirect effect = −0.08, CI [−0.23, 0.09]) and their sense of competence in teaching (indirect effect = 0.14, CI [−0.001, 0.18]). Additionally, the interns’ autonomous motivation in school was found to fully mediate the association between the teacher-mentor’s support and their sense of self-actualization (indirect effect = 0.37, CI [0.18, 0.51]) and their sense of competence in teaching (indirect effect = 0.28, CI [0.15, 0.42]). However, interns’ autonomous motivation in school did not mediate the association between teacher-mentor’s support and burnout in school (indirect effect = −0.16, CI [−0.21, 0.04]).

Multigroup analyses were conducted to examine whether there are differences between Bedouin Arabs and Jews in the proposed mediation model. To this end, a comparison was conducted between the two models: the first model allows free calculation of the association between the variables in each group; in the second model, the association between the variables in the two groups was forced to be equal. Then, the free model and the constrained model were compared to investigate whether the mediation model is equivalent for Bedouin Arabs and Jews. In accordance with H3, the findings show no significant differences between the two models, which indicates that there are no significant differences between Bedouin Arabs and Jews in the association between the variables in the proposed mediation model (Delta chi-square (15) = 5.23, *p* = 0.54).

### Testing hypothesis 2: Mediation model T2

To corroborate the second research hypothesis (H2), the hypothesized model was examined by means of structural equation modeling (SEM), using AMOS 21 software. The model includes the variables “LC facilitator need and exploration support” (T2) and “teacher-mentor need and exploration support” (T2) as independent exogenous latent variables. The variables “autonomous motivation in the LC” (T2), and “autonomous motivation in school” (T2) are mediating variables, while the variables “satisfaction with the LC” (T2), “positive feelings in the LC” (T2), “self-actualization” (T2), “sense of competence in teaching” (T2), and “sense of burnout in teaching” (T2) are dependent variables. In order to conduct a controlled examination of the effects of the variables, the variance of the variables at T2 were controlled by the variance of the variables at T1.

**Figure 2:**
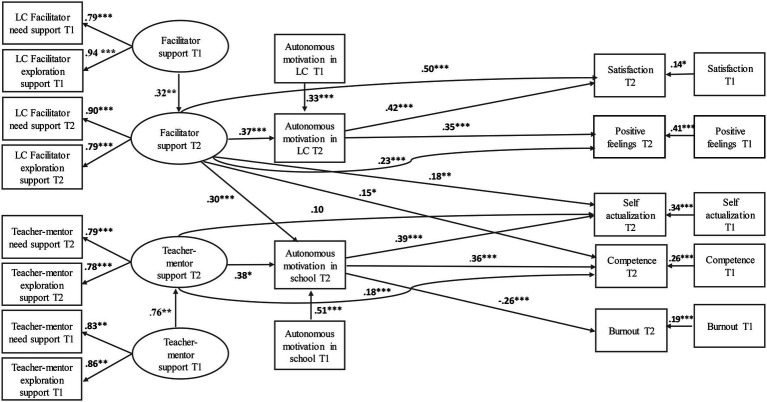
Structural equation modeling analysis results – T2. *p<0.05; **p<0.01; ***p<0.001.

The goodness of fit results show goodness of fit between the hypothesized model and the data: χ^2^ = 575.01, df = 174, *p* < 05; TLI = 0.92; CFI = 0.96; RMSEA = 0.07. Then, the associations in the hypothesized model were examined. Figure 2 presents the results for the hypothesized structural model. In addition, Table 5 shows the raw and standardized data, t-statistic value, and their significance. Figure 2 shows the results.

**Table 5:** Results of the mediation model with T1 and T2 variables.

	Beta	*B*	SE	*t*	*P*
LC facilitator support (T2) → Autonomous motivation in workshop T2	0.373	0.598	0.092	6.476	<0.001
Teacher-mentor support (T2) → Autonomous motivation in teaching T2	0.380	0.341	0.075	4.612	0.038
Autonomous motivation in workshop (T1) → Autonomous motivation in workshop T2	0.329	0.291	0.051	5.668	<0.001
Autonomous motivation in teaching (T1) → Autonomous motivation in teaching T2	0.513	0.480	0.069	6.916	<0.001
LC facilitator support (T2) → Autonomous motivation in teaching T2	0.297	0.304	0.070	4.359	<0.001
LC facilitator support (T2) → LC facilitator need support (T2)	0.792	0.729	0.070	10.416	<0.001
LC facilitator support (T2) → LC facilitator exploration support (T2)	0.782	1.375	0.133	10.415	<0.001
Autonomous motivation in workshop (T2) → Satisfaction with workshop T2	0.423	0.342	0.042	8.219	<0.001
Autonomous motivation in workshop (T2) → Positive feelings in workshop T2	0.351	0.337	0.054	6.204	<0.001
Teacher-mentor support (T2) → Teacher-mentor exploration support (T2)	0.792	1.151	0.303	11.194	<0.001
Teacher-mentor support (T2) → Teacher-mentor need support (T2)	0.896	0.867	0.077	11.195	<0.001
Autonomous motivation in teaching (T2) → Burnout T2	−0.263	−0.481	0.103	−4.654	<0.001
Autonomous motivation in teaching (T2) → Self-actualization T2	0.394	0.471	0.065	7.255	<0.001
Autonomous motivation in teaching (T2) → Sense of competence in teaching T2	0.365	0.326	0.051	6.380	<0.001
Teacher-mentor support (T2) → Sense of competence in teaching T2	0.185	0.159	0.055	2.920	0.004
LC facilitator support (T2) → Satisfaction with workshop T2	0.503	0.652	0.073	8.873	<0.001
LC facilitator support (T2) → Positive feelings in workshop T2	0.235	0.361	0.096	3.773	<0.001
Satisfaction with workshop (T1) → Satisfaction with workshop T2	0.146	0.121	0.047	2.574	0.010
Positive feelings in workshop (T1) → Positive feelings in workshop T2	0.410	0.315	0.046	6.796	<0.001
Self-actualization (T1) → Self-actualization T2	0.340	0.278	0.050	5.600	<0.001
Burnout T1 → Burnout T2	0.197	0.123	0.050	2.488	0.013
Sense of competence in teaching (T1) → Sense of competence in teaching T2	0.257	0.249	0.065	3.819	<0.001
LC facilitator support (T2) → Sense of competence in teaching T2	0.152	0.139	0.060	2.330	0.020
LC facilitator support (T2) → Self-actualization T2	0.180	0.221	0.069	3.196	0.001
Teacher-mentor support (T2) → Self-actualization T2	0.106	0.122	0.068	1.796	0.073
LC facilitator support (T1) → LC facilitator support (T2)	0.32	0.31	0.08	3.79	<0.001
Teacher-mentor support (T1) → Teacher-mentor support (T2)	0.76	0.60	0.07	8.64	<0.001

A significant positive association was found between the LC facilitator’s support for the new teachers (T2) and their autonomous motivation in the LC (T2), which in turn predicted their satisfaction with the LC and their positive feelings in the LC (T2). Additionally, a direct significant positive association was found between the LC facilitator’s support for the new teachers (T2) and their satisfaction with the LC and their positive feelings. Consequently, and in accordance with H2, the new teachers’ autonomous motivation in the LC (T2) was found to partially mediate the association between the LC facilitator’s support for the teachers (T2) and their satisfaction with and positive feelings in the LC (T2).

It further emerges that there is a significant positive association between the LC facilitator’s support and the teacher-mentor’s support for the new teachers (T2) and their autonomous motivation in school (T2), which in turn significantly positively predicts sense of self-actualization (T2), and sense of competence in teaching (T2), and significantly negatively predicts sense of burnout in school (T2). Additionally, a direct significant positive association was found between the LC facilitator’s and teacher-mentor’s support for the teachers and their sense of self-actualization in school, and their sense of competence in teaching (T2).

Consequently, and in accordance with H2, the new teachers’ autonomous motivation in school (T2) was found to partially mediate the association between the teacher-mentor’s support (T2), and their sense of self-actualization, but did not mediate the association between the LC facilitator’s support and self-actualization (LC facilitator indirect effect = 0.03, CI [−0.12, 0.08]; teacher-mentor support indirect effect = 0.22, CI [0.10, 0.34]). In addition, the new teachers’ autonomous motivation in school (T2) was found to partially mediate the association between the teacher-mentor’s support (T2) and their sense of competence, but did not mediate the association between the LC facilitator’s support and sense of competence (LC facilitator indirect effect = 0.02, CI [−0.24, 0.09]; teacher-mentor support indirect effect = 0.18, CI [0.06, 0.23]). Furthermore, the new teachers’ autonomous motivation in school (T2) was not found to be a significant mediator in the association between the LC facilitator’s and teacher-mentor’s support, and their sense of burnout (LC facilitator indirect effect = −0.03, CI [−0.29, 0.14]; teacher-mentor support indirect effect = −0.08, CI [−0.17, 0.16]) (T2).

The associations between the research variables at T2 were found above and beyond the variance of the variables at T1. The significance of this finding is that the continuation LCs and the teacher-mentor’s support in the second year had a unique effect, beyond the effects of the LCs and the teacher-mentor’s support in the first year.

The results of the mediation model with T1 and T2 variables are shown in Table 5.

In addition, multigroup analyses were conducted to examine whether there are differences between Bedouin Arabs and Jews in the proposed mediation model (see detailed analysis above). In accordance with H3, the findings show no significant differences between Bedouin Arabs and Jews in the association between the variables in the proposed mediation model (Delta chi-square (20) = 10.23, *p* = 0.79).

### Testing an alternative model

To examine whether the research hypotheses best describe the association between the variables, an alternative model was investigated. The model includes the variables “sense of burnout in teaching” (T2), “satisfaction with the LC” (T2), “positive feelings in the LC” (T2), “self-actualization” (T2), and “sense of competence in teaching” (T2) as independent variables, The variables “autonomous motivation in the LC” (T2), and “autonomous motivation in school” (T2) are mediating variables, while the variables “LC facilitator need and exploration support” (T2) and “teacher-mentor need and exploration support” (T2) as dependent latent variables. The variance in variables in T2 was controlled by the variance in T1 variables.

The goodness of fit results placed emphasis on bad model fit indices: χ^2^ = 912.18, df = 174, *p* < 0.05; TLI = 0.49; CFI = 0.63; RMSEA = 0.14.

Analyses showed a significant positive association between positive feelings in the LC (T2) and autonomous motivation in the LC (T2) (β = 0.56, t = 5.93, *p* < 0.001), which in turn was positively associated with the LC facilitator’s support for the new teachers (T2) (β = 0.43, *t* = 4.71, *p* < 0.001). The association between satisfaction in the LC (T2) and autonomous motivation in the LC (T2) (β = −0.18, *t* = −1.25, *p* = 0.21) was not significant. Additionally, the associations between teachers’ burnout (T2) (β = −0.15, *t* = −0.09, *p* = 0.07), competence (β = 0.03, *t* = 0.16, *p* = 0.87), and self-actualization (T2) (β = 0.34, *t* = 1.59, *p* = 0.10), and autonomous motivation in school (T2), was not significant. Autonomous motivation in school (T2) was positively associated with teacher-mentor support (T2) (β = 0.24, *t* = 2.76, *p* = 0.006). All T2 variables were controlled by T1 variables. Together they strengthened the hypothesized mediation model.

## Discussion

The present study enabled a simultaneous examination of two induction programs over a 2-year period – LCs in workshop format that took place at a college of education (in the internship year and the year after internship), and mentoring by a teacher-mentor, which took place in the school. The study examined the unique contributions of the LC facilitators’ and teacher-mentors’ need support and exploration support to predicting outcomes associated with the BTs’ functioning in the LC and the school, and the mediating role of autonomous motivation, in each of the 2 years. The study also examined whether participation in an LC and support from a teacher-mentor in the second year have a unique contribution beyond that of the first year. The findings support the research hypotheses, as presented below.

### The importance of multiple induction programs: LC facilitator and teacher-mentor support

The results show a significant positive association in both years between the LC facilitator’s support for the BTs and their autonomous motivation in the LC, which in turn predicted their sense of satisfaction with and positive feelings in the LC. The BTs’ autonomous motivation in the LC partially mediated the association between the LC facilitator’s support and their sense of satisfaction with and positive feelings in the LC. These effects were found above and beyond the effects of the teacher-mentors’ support.

In addition, a significant positive association was found between the LC facilitators’ support and BTs’ autonomous motivation in school, which in turn influenced positive outcomes among them. At T1, the interns’ autonomous motivation in school fully mediated the association between the LC facilitator’s support and their sense of self-actualization, but did not mediate the association between the LC facilitator’s support and their sense of burnout and sense of competence. At T2, the new teachers’ autonomous motivation in school was not found to significantly mediate the association between the LC facilitator’s support and their sense of self-actualization, competence, and burnout.

It therefore appears that the more the BTs perceive the LC facilitator as supporting their needs and exploration, the better they function in the LC and in school. The findings reinforce the findings of previous studies indicating that supporting the needs of experienced teachers, and experiences of need satisfaction, are associated with a range of positive outcomes, such as autonomous motivation in teaching (Eyal and Roth, 2011), self-actualization, and investment (Kaplan and Madjar, 2017). Autonomous motivation in teaching was found to be associated with sense of self-actualization, and negatively associated with sense of emotional burnout among teachers (Roth et al., 2007). Among BTs, too, need support was found to lead to autonomous motivation in teaching, job commitment, self-actualization, investment, reduced burnout, and more (Fernet et al., 2016; Kaplan, 2021b). The findings join those of studies proving the efficacy of BT support workshops, which in the present study function as LCs, both from an SDT perspective (e.g., Kaplan et al., 2016; Kaplan, 2021b), and other theories (e.g., Ingersoll and Strong, 2011; Fresko and Nasser-Abu Alhija, 2014).

The findings indicate the contribution of LCs to BTs. According to the research literature, LCs of teachers contribute to promoting the participants’ wellbeing (Owen, 2016), and foster knowledge, and new skills and practices (Fresko and Nasser-Abu Alhija, 2014), which, according to SDT, are associated with sense of competence, as well as the professional identity construction of BTs (Hammer-Budnaro, 2018). The present study indicates that LCs also contribute to autonomous motivation in teaching, which in turn predicts sense of competence and self-actualization (which is associated with identity construction).

Numerous studies focus on the importance of LCs for BTs that take place in schools, with reference to aspects such as the principal’s leadership, mentoring, and school climate (e.g., Wynn and Brown, 2008). The present study examines LCs that take place outside the schools, and indicates their contribution. Fresko and Nasser-Abu Alhija (2014) conducted a similar examination, and found that the main contribution of the LCs was in the affective aspect. The LCs served as a safe place in which the teachers could express their feelings and frustrations without fear of being judged. The present study reinforces this aspect, and indicates the contribution of the LCs to supporting psychological needs and promoting experiences of need satisfaction, which are affective in essence. The induction process is complex and can impact the emotional resources of BTs, as found in previous studies (Clandinin et al., 2015; Voss et al., 2017; Kaplan, 2021a). Consequently, their psychological needs should be supported (Fresko and Nasser-Abu Alhija, 2014).

Additional unique effects were found that refer to the teacher-mentors’ support. A significant positive association was found in both years between the teacher-mentors’ support and the BTs’ autonomous motivation in school. At T1, the interns’ autonomous motivation in school was found to fully mediate the association between the teacher-mentors’ support and the interns’ sense of self-actualization and competence, but not burnout. At T2, the new teachers’ autonomous motivation in school was found to partially mediate the association between the teacher-mentors’ support and the new teachers’ sense of self-actualization and competence, but not burnout. These effects were found above and beyond the effects of the LC facilitators’ support.

Mentoring is one of the most widely acknowledged and extensively researched induction programs, and numerous positive outcomes are attributed to teacher-mentor support among BTs (Richter et al., 2013; Alegado and Soe, 2021; Ewing, 2021). Very few SDT-based studies examined the effects of the mentors’ support among BTs (Kaplan and Israel, 2020, Unpublished manuscript, See footnote 2; Burger et al., 2021; Kaplan, 2021b). The present study joins these studies, and shows that the teacher-mentors’ support has unique positive effects among BTs in various stages of their professional development.

There are a variety of different paradigms pertaining to optimal mentoring relationships (Burger et al., 2021). The present study focuses on SDT as a mentoring paradigm that supports BTs’ growth processes (Orland-Barak and Wang, 2020) by means of psychological need support. This kind of mentoring characterizes a paradigm that Burger et al. (2021) term “constructivist-oriented mentoring.” In both studies, need supportive mentoring promoted sense of self-actualization sense of competence by satisfying the need for autonomy among mentors (in the present case by means of autonomous motivation). This mentoring outlook places the relationship between the teacher-mentor and the mentored teacher at the center. According to this approach, the mentoring relationship facilitates partnership, mutual support, and experiences of psychological need satisfaction. The relationship is founded on trust and on creating a safe dialogical space for discussing relevant internal content – the content in the sessions is jointly determined by the mentor and mentee (Kaplan and Israel, 2020, Unpublished manuscript, See footnote 2).

In Israel, there are tensions between Ministry of Education policies concerning LCs and the mentors’ role, and the authentic needs of interns and new teachers. Work with BTs on their professional development is typified by a top-down directive approach. Thus, the LCs are mandatory and include evaluation of the interns by their mentors and the school principal (Fresko and Nasser-Abu Alhija, 2014; Ford, 2017). Much of the content in the LCs is dictated by policymakers (e.g., discussions on evaluation processes and their significance for the teachers, or on the place of the mentor as an evaluator of the intern’s teaching while being a source of support). According to SDT, directives and pressures from policymakers, evaluation, judgment, comparison of achievements between teachers, and so on, can create an experience of control, impair autonomous motivation, and encourage extrinsic goals (Ryan and Deci, 2020). The study indicates that even in the current situation, the more teachers experience need support from the LC facilitator and teacher-mentor, the higher their sense of autonomous motivation, sense of competence, and sense of self-actualization. It therefore appears that need support is essential and crucial in the process of teachers’ induction into school and teaching.

The findings indicate that the teachers’ functioning in school is influenced by the teacher-mentor’s support, which takes place in the school, as well as the LC facilitator’s support, which takes place outside the school. Thus, the more BTs perceived both the LC facilitator and the teacher-mentor as need supportive, the more they expressed better functioning as teachers. The effects of the LC on teachers’ functioning in school can be explained by the fact that the LC engages with content that is relevant to them. They raise issues and events for discussion that are significant to their work in school (Hammer-Budnaro, 2018). They pass their learning from these discussions with their fellow teachers in the LC and the facilitators onto the school.

The findings pertaining to the positive contributions of the LC facilitators’ and teacher-mentors’ support reinforce the findings of a previous study (Kaplan, 2021b), which found that these two sources of support positively contribute to predicting positive outcomes, both in the LC and the school. The previous study also found that autonomous motivation in teaching was predicted both by the LC facilitators’ and the teacher-mentors’ support, which in turn predicted sense of competence, investment, and sense of self-actualization.

Previous research shows that BTs who received support from multiple induction programs experience positive outcomes. For example, they are less likely to transfer to other schools, or leave the teaching profession (Smith and Ingersoll, 2004). The present study indicates additional contributions of combining a number of induction programs. Thus, unique contributions to the BTs’ successful functioning in school were found in each of the sources of support examined in the study.

### The unique contributions of the LC facilitators’ and teacher-mentors’ support during the 2 years of the study

The results at T2 were found above and beyond the effects found at T1. The significance of this finding is that the LC and mentoring in the second year had a unique contribution, above and beyond the effects of the LC and mentoring in the first year.

The findings indicate the unique contribution of each of the two induction programs, as well as their cumulative effect during the 2 years of the intervention. It is important to note that these two induction programs engage with the same content world – induction into teaching. Consequently, there was a possibility that the teachers might experience the support they received in the second year as ineffective compared to the first year, which was not the case in the present study.

These findings indicate that the new teachers come to the second year equipped with inner resources, which increasingly grow during the year. In the present study, these resources are autonomous motivation, sense of competence, and sense of self-actualization, which are influenced by support from the LC facilitators and teacher-mentors during the 2 years. The development of these inner resources is particularly important for BTs who face numerous difficulties (De Neve and Devos, 2017), and need-frustration during this period (Kaplan, 2021a).

According to Vansteenkiste and Ryan (2013), motivational processes that develop as a result of need support (e.g., sense of relatedness, sense of competence, sense of choice, autonomous motivation) serve as sources of inner resilience that can buffer the negative effects or dysfunction that can be bound up in situations of crisis or difficulty, which BTs face. The researchers argue that people who act from a sense of autonomy regulate their behavior by establishing personal interest, authentic preferences, and inner values. Such people are more open to new experiences, and equipped with sources of inner resilience that can help them in their personal and professional life. Thus, the processes the teachers undergo in the LC or mentoring can moderate the negative implications of the difficulties they encounter during the induction period in school and in teaching. According to the researchers, these abilities are supported by the individual’s awareness of their abilities and inner resources (which are promoted by exploration support in the present study).

The finding whereby the LC and mentoring in the second year have a unique contribution beyond the contribution of the support the teacher received in the first year, has implications pertaining to the considerations of policymakers and principals concerning customary professional development processes in Israel and around the world. Teachers’ professional development processes are central in the thinking of educational policymakers. There is a worldwide trend of shifting away from short-term interventions towards a broader perception of professional development as an ongoing process that places emphasis on teachers’ needs and on their autonomy. This approach emphasizes reflective-self learning, and collaborative processes between teachers that take place in an LC that responds to the human need for relatedness (Mikulincer and Parzachevsky Amir, 2020). The present study’s findings support this trend. They support a processual perception of professional development carried out in LCs that provides a response to the teachers’ psychological needs, and promotes reflective processes by means of need support and exploration support.

The information on the efficacy of professional development processes over two consecutive years has economic implications as well, which policymakers seeking to promote programs with proven efficacy take into consideration. Additionally, it appears that teachers benefit from this support during the 2 years, an important consideration with reference to effective use of the teacher’s time, especially during the demanding period of entering the teaching profession and the school.

### Identity construction processes among beginning teachers

The study’s findings indicate the connection between need support and identity construction processes, as implied by the finding whereby need support and exploration support promote autonomous motivation in teaching, which in turn also predicts sense of self-actualization. A study on teacher education indicates the importance of identity construction during teachers’ professional learning (Beauchamp and Thomas, 2009). Assor (2012) conceptualizes the need for autonomy as the need to fulfill and formulate authentic values, goals, and interests that will guide the teacher’s professional path. A need supportive environment is necessary to promote this identity construction process (La Guardia, 2009).

La Guardia (2009) argues that although the nucleus of a person’s identity is solidified in adulthood, at different times in their life their identity can undergo reorganization. Life transitions (developmentally normative or imposed) require people to take on new challenges, consider how to integrate new activities, roles, and relationships, and ultimately grapple with how they conceive themselves. Thus, the induction period is a normative professional development stage that can lead to fundamental questions of identity (Kaplan et al., 2016). The teachers’ difficulties can raise questions concerning their suitability for the profession, and their desire to remain in it. A need supportive and exploration supportive environment can also promote identity construction processes that reinforce teachers’ autonomous motivation in teaching.

### The study’s cultural context

In accordance with H3, the findings in both time measurements show no significant differences between Bedouin Arabs and Jews in the association between the variables in the proposed mediation model. The findings support the applicability of SDT in different cultural groups, and its universality claim whereby all people benefit from basic need satisfaction regardless of their cultural background (Vansteenkiste and Ryan, 2013; Ryan and Deci, 2017, 2020). They refute the arguments of cross-cultural researchers who challenge the universality of SDT (e.g., Iyengar and DeVoe, 2003; Liu and Flick, 2019), claiming that the need for autonomy is a Western ideal and is not important in traditional Eastern cultures, which emphasize values of conformity and social harmony, rather than values of individuality and independence. According to them, as opposed to the findings of studies conducted in Western cultures, no positive effects of autonomy support will be found in a collectivist society.

According to SDT, psychological needs are universal and innate, and need support is expected to lead to autonomous motivation in different cultural groups (Deci and Ryan, 2000) despite possible differences in the level of need satisfaction (Chirkov et al., 2003). Studies comparing populations from different cultural backgrounds found that the psychological mechanisms connecting need support and motivation are similar across different cultural contexts (Chen et al., 2015; Ginevra et al., 2015).

The results of the present study underscore SDT’s universality claim (Ryan and Deci, 2017), and are consistent with the findings of SDT-based studies conducted in countries with Eastern collectivist societies, e.g., South Korea (Jang et al., 2009), Turkey (Chirkov et al., 2003), China, Hong Kong, and Japan (Nalipay et al., 2020) and Bedouin society (e.g., Kaplan et al., 2014; Kaplan, 2018).

However, previous studies conducted in Bedouin society that examined need satisfaction among teachers, primarily focused on in-service teachers (e.g., Katz and Cohen, 2018), and pre-service teachers (e.g., Kaplan and Madjar, 2017). The research population in the present study – interns and new teachers during their induction period – has received very little research attention from an SDT perspective (e.g., Kaplan, 2021b). The present study contributes to current knowledge in this respect.

### Practical implications

The study indicates the importance of combining multiple induction programs during the induction period. However, in any program aiming to support BTs it is important to create conditions that support their psychological needs. Thus, autonomy-based mentoring programs (Kaplan and Israel, 2020, Unpublished manuscript, See footnote 2) can be built into different mentoring models (e.g., peer group mentoring, dyadic mentoring). In LCs, relationships typified by need support should be formed between the facilitator and the participants, and between the participants themselves.

Another recommendation is for induction programs to continue over several years. The present study focused on 2 years during which the teachers acquired inner resilience resources. It is important for the different programs to be coherent in terms of their outlook. The present study examined the SDT approach, a recommended guiding approach in various induction programs.

The principles of SDT include three core practices: support for autonomy, support for competence, and support for relatedness, which are based on previous research (Assor et al., 2002; Reeve, 2006; Kaplan and Madjar, 2017). In a population of BTs, psychological need support includes a range of facilitator and mentor behaviors. Relatedness support, for example, includes strengthening acquaintance, displaying caring and interest in the teacher’s activities, available assistance, and a reflective dialogue that facilitates sharing of feelings. Competence support includes providing structure, i.e., a roadmap of the school, its requirements and routines, clarification of expectations, setting optimal challenges, and providing informative, non-judgmental feedback. And autonomy support includes discussing authentic events for the BT, reference to their unique views and opinions, enabling choice, encouraging initiatives, involvement in decision making, providing a rationale, reinforcing relevance, and avoiding coercion (e.g., using controlling language, setting demands without explanation, etc.).

### Limitations and future directions

The present study has several limitations. The first pertains to the research tools, which are based on self-report questionnaires as the sole source of information. Some scholars question the reliability of respondents’ self-perceptions (Fulmer and Frijters, 2009), while others claim that in the educational context, it is the participants’ subjective perceptions that influence outcomes (Madjar et al., 2013). Additionally, self-reports can reflect a response bias (e.g., in situations of social desirability), which may be characteristic of BTs from a collectivist culture (Al-Krenawi, 2010).

In future studies, it is important to incorporate additional research tools. For example, a qualitative study employing interviews to examine how teachers experience the LC in the second year, and its value in their view compared to the LC in the first year, from a psychological needs perspective. This direction has been proposed by Ryan and Deci (2020) who suggest using a qualitative research design in an SDT framework in order to obtain a more detailed picture of experiences, practices, and motivations of teachers in schools and practical SDT frameworks.

Another limitation is that the study examined psychological need support and exploration support, and experiences of need satisfaction, and not situations of need thwarting and need frustration. Nowadays, there is a research-based perception whereby psychological need satisfaction plays a significant role in people’s growth and wellbeing, while need frustration plays a significant role in predicting negative reactions (Ryan and Deci, 2000; Vansteenkiste and Ryan, 2013). Various studies have provided evidence for the distinction between need support and need thwarting, and need-satisfaction and need-frustration (van der Kaap-Deeder et al., 2020). Following this line of research, in the present study at both T1 and T2, the interns’ and new teachers’ autonomous motivation in school did not mediate the support they received from the teacher-mentors and the LC facilitators and their sense of burnout. Thus, future research should also examine this range of aspects. Investigation of these aspects is important in light of the extensive research describing BTs’ negative experiences during their induction period, including situations of psychological need suppression and negative reactions, as found in qualitative studies (e.g., Kaplan, 2021a).

Another limitation pertains to analysis of the research data. A manifest factor model was used to assess the hypothesized mediation, rather than latent factor model. The reason for using manifest variables was due to the large number of predictors included in the model, and also to keep the recommended ratio of 10:1 between sample size and number of predictors.

Additionally, the present study focuses on LCs outside the school. In a school as a community there are various figures who can influence the absorption process of BTs: the principal, experienced teachers, administrators, parents, and others. Consequently, another research direction could be an examination of school-based LCs as a support resource for new teachers, in addition to the mentor’s role as a source of support, all from an SDT perspective, of course.

In summary, the literature in Israel and around the world contains a wealth of studies describing the difficulties of BTs (Mansfield et al., 2014; Flores, 2017). One of the most common metaphors in the literature refers to the transition from training to teaching as a “reality shock” (e.g., Voss et al., 2017). The present study indicates the importance of support systems for BTs, in the present case an LC and a mentor’s support, which by supporting their needs and facilitating experiences of need satisfaction can prevent or moderate this sense of reality shock. Experiences of need support become resilience resources that continue to accompany the teachers during a second year of receiving various kinds of support, and, it may be assumed, throughout their professional life as well. The study demonstrates the importance of translating the theory into practice, and constructing environments for BTs that support their needs and help their optimal absorption into the school and the teaching profession.

## Data availability statement

The original contributions presented in the study are included in the article/supplementary material, further inquiries can be directed to the corresponding author.

## Ethics statement

The studies involving human participants were reviewed and approved by Research Authority of Kaye College. The patients/participants provided their written informed consent to participate in this study.

## Author contributions

HK performed the study conception, design, material preparation, data collection, and analysis, and wrote the manuscript.

## Conflict of interest

The author declares that the research was conducted in the absence of any commercial or financial relationships that could be construed as a potential conflict of interest.

## Publisher’s note

All claims expressed in this article are solely those of the authors and do not necessarily represent those of their affiliated organizations, or those of the publisher, the editors and the reviewers. Any product that may be evaluated in this article, or claim that may be made by its manufacturer, is not guaranteed or endorsed by the publisher.

## Footnotes

^1^The term "beginning teachers" (BTs) refers to "interns" (usually in their fourth year of study) and "new teachers" (first year after internship). All three terms will be used in this article according to context.

^2^Kaplan, H., and Israel, V. (2020). "Autonomy-based mentoring: the ABC-C mentoring model (autonomy/belongingness/competence/community)," in Kaye academic College of Education, growth recourses Kaye induction unit result of Package 1, led by Kaye College, within the international Promentors Project, EU Erasmus+ Program. (Unpublished manuscript).
